# Supplementary material for: Tumor-Infiltrating Lymphocyte Level Consistently Correlates with Lower Stiffness Measured by Shear-Wave Elastography: Subtype-Specific Analysis of Its Implication in Breast Cancer
Source: Cancers (Basel). 2024 Mar 22;16(7):1254. doi: 10.3390/cancers16071254 (PMC11011118; doi:10.3390/cancers16071254)

Supplementary Figure S1. Representative image of shear-wave elastography

A 64-year-old woman with a pathologically proven invasive ductal carcinoma. Shear-wave elastography (upper image) and B-mode image (lower image) reveal a 23-mm irregular mass displaying a red, heterogeneous elasticity pattern. To quantify the elasticity of the lesion, a region of interest (ROI) was placed over the stiffest portion of the mass (indicated by a circle), from which mean, minimum, maximum, and standard deviation of elasticity values were computed in kilopascals (kPa). Additionally, the elasticity ratio of the mass compared to the surrounding fat tissue was determined by placing a second ROI over the adjacent fatty tissue area (marked by a dotted circle).

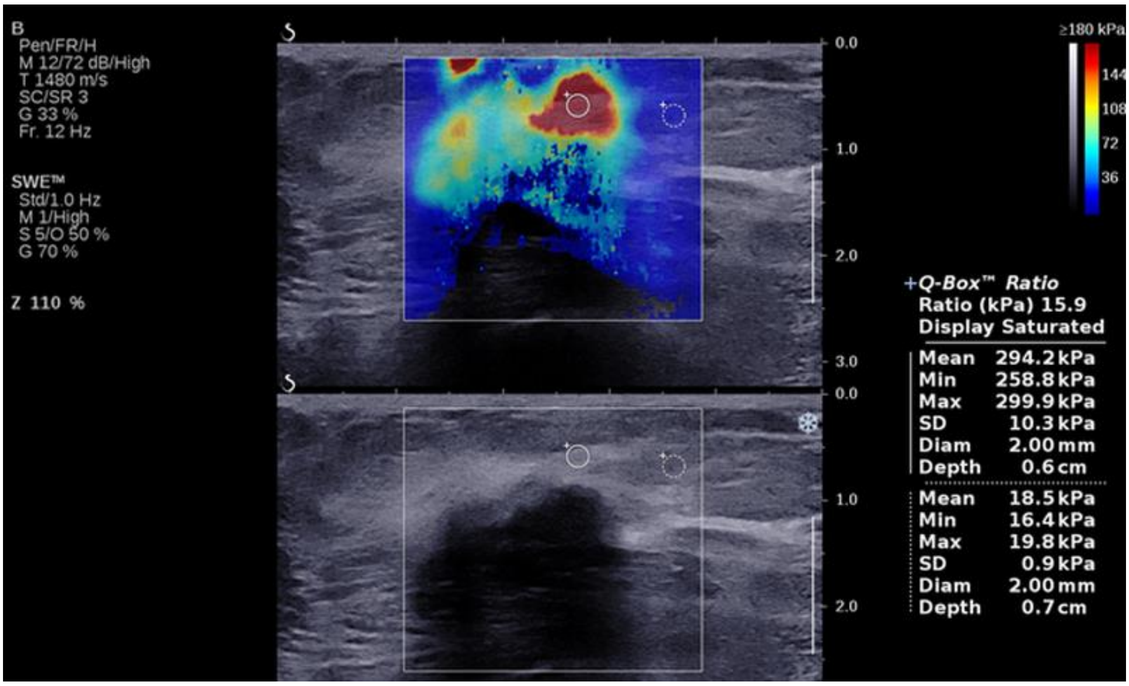

Supplementary Figure S2. Forest plots of linear regression analysis assessing the association between tumor elasticity and clinicopathologic parameters across different tumor subtypes

HR+HER2-, hormone receptor-positive, HER2-negative; BC, breast cancer; TNBC, triple-negative breast cancer; DCIS, ductal carcinoma in situ; TSR, tumor stroma ratio; TIL, tumor-infiltrating lymphocytes; LI, labeling index; NG, nuclear grade; HG, histologic grade; LVI, lymphovascular invasion; LN, lymph node; EIC, extensive intraductal component (>25%)

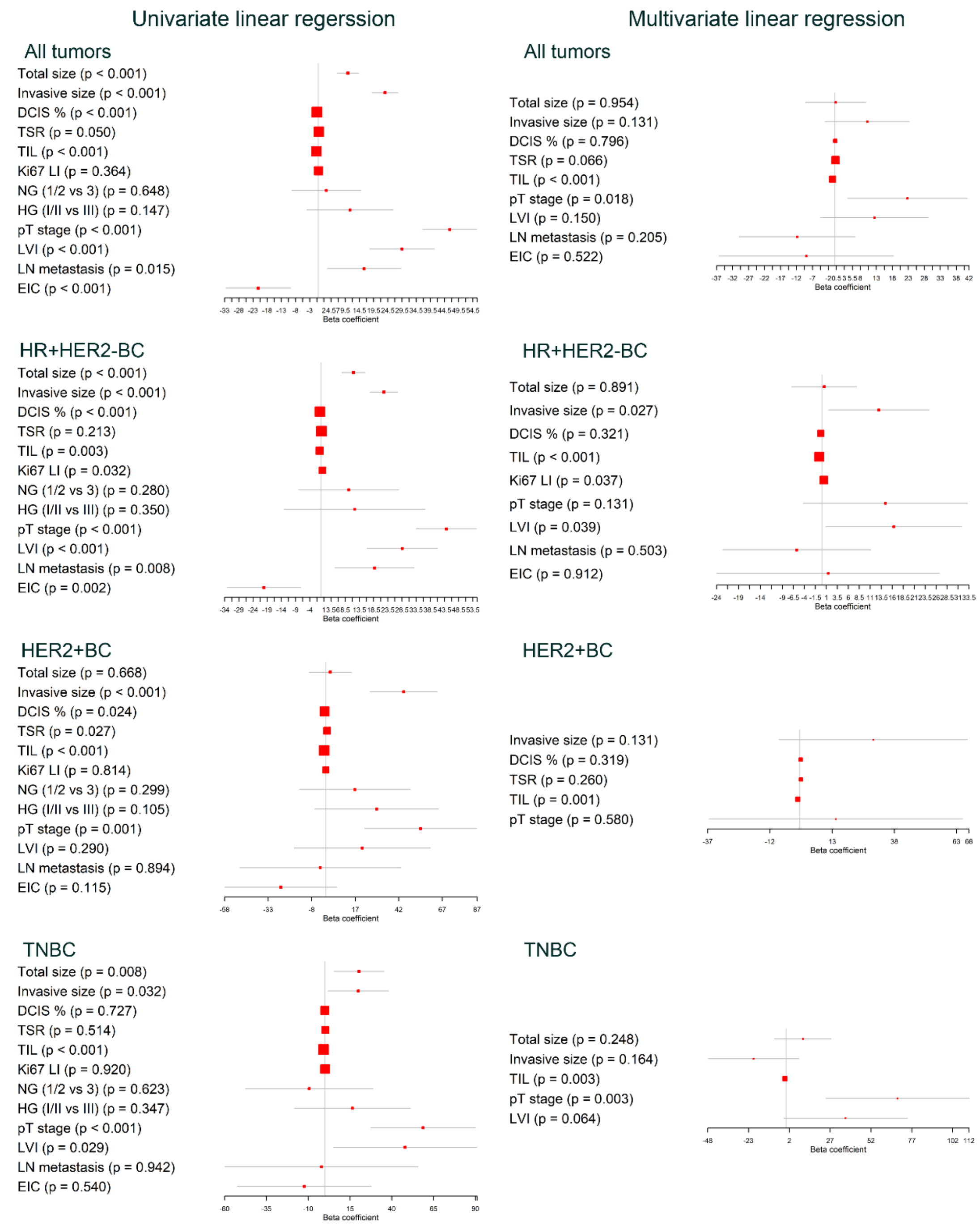

Supplement: Supplementary file 1 [file cancers-16-01254-s001.zip › supplementary figures.pdf]
