# Supplementary material for: Tumor-Infiltrating Lymphocyte Level Consistently Correlates with Lower Stiffness Measured by Shear-Wave Elastography: Subtype-Specific Analysis of Its Implication in Breast Cancer
Source: Cancers (Basel). 2024 Mar 22;16(7):1254. doi: 10.3390/cancers16071254 (PMC11011118; doi:10.3390/cancers16071254)
Supplement: Supplementary file 1 [file cancers-16-01254-s001.zip › supplementary tables.pdf]

Supplementary Table S1. Correlation matrix of different tumor subtypes (*r* coefficient and *p*-value)

	All tumor			HR+HER2-BC			HER2+BC			TNBC		
	E_{mean}	E_{max}	E_{min}	E_{mean}	E_{max}	E_{min}	E_{mean}	E_{max}	E_{min}	E_{mean}	E_{max}	E_{min}
Invasive size	0.32 (*p*<0.001)	0.34 (*p*<0.001)	0.21 (*p*<0.001)	0.32 (*p*<0.001)	0.34 (*p*<0.001)	0.19 (*p*<0.001)	0.41 (*p*<0.001)	0.42 (*p*<0.001	0.37 (*p*<0.001)	0.26 (*p*=0.026)	0.25 (*p*=0.032)	0.26 (*p*=0.028)
Total size	0.18 (*p*<0.001)	0.19 (*p*<0.001)	0.11 (*p*=0.002)	0.21 (*p*<0.001)	0.21 (*p*<0.001)	0.12 (*p*=0.003)	0.04 (*p*=0.69)	0.04 (*p*=0.670)	0.04 (*p*=0.690)	0.25 (*p*=0.037)	0.31 (*p*=0.008)	0.19 (*p*=0.110)
TSR	0.09 (*p*=0.035)	0.08 (*p*=0.05)	0.07 (*p*=0.078)	0.05 (*p*=0.260)	0.06 (*p*=0.210)	0.05 (*p*=0.260)	0.29 (*p*=0.0074)	0.24 (*p*=0.027)	0.32 (*p*=0.002)	0.1 (*p*=0.510)	0.09 (*p*=0.510)	0.01 (*p*=0.920)
HG	0.06 (*p*=0.072)	0.06 (*p*=0.081)	0.03 (*p*=0.380)	0.06 (*p*=0.160)	0.07 (*p*=0.086)	0.02 (*p*=0.550)	0.17 (*p*=0.096)	0.17 (*p*=0.092)	0.14 (*p*=0.170)	0.14 (*p*=0.240)	0.11 (*p*=0.350)	0.1 (*p*=0.390)
NG	0.02 (*p*=0.480]	0.02 (*p*=0.530)	0 (*p*=0.960)	0.04 (*p*=0.320)	0.05 (*p*=0.190)	0.01 (*p*=0.820)	0.09 (*p*=0.35)	0.1 (*p*=0.300)	0.06 (*p*=0.540)	-0.03 (*p*=0.780)	-0.06 (*p*=0.620)	-0.07 (*p*=0.550)
Ki67 LI	0.04 (*p*=0.230)	0.03 (*p*=0.360)	0.1 (*p*=0.004)	0.08 (*p*=0.055)	0.09 (*p*=0.032)	0.18 (*p* <0.001)	0.02 (*p*=0.88)	0.02 (*p*=0.810)	0.02 (*p*=0.810)	0.07 (*p*=0.580)	0.01 (*p*=0.920)	0.08 (*p*=0.530)
DCIS %	-0.13 (*p*<0.001)	-0.15 (*p*<0.001)	-0.09 (*p*=0.011)	-0.11 (*p*=0.005)	-0.14 (*p*<0.001)	-0.07 (*p*=0.066)	-0.23 (*p*=0.021)	-0.22 (*p*=0.024)	-0.18 (*p*=0.071)	-0.07 (*p*=0.580)	-0.04 (*p*=0.730)	-0.07 (*p*=0.550)
TIL	-0.18 (*p*<0.001)	-0.2 (*p*<0.001)	-0.12 (*p*=0.001)	-0.11 (*p*=0.005)	-0.12 (*p*=0.003)	-0.05 (*p*=0.18)	-0.34 (*p*=0.001)	-0.33 (*p*<0.001)	-0.31 (*p*=0.001)	-0.35 (*p*=0.002)	-0.38 (*p*<0.001)	-0.3 (*p*=0.010)

HR+HER2-, hormone receptor-positive, HER2-negative; BC, breast cancer; TNBC, triple-negative breast cancer; E_{mean}, mean elasticity; E_{max}, maximal elasticity; E_{min}, minimal elasticity; TSR, tumor stroma ratio; HG, histologic grade; NG, nuclear grade; LI, labeling index; DCIS, ductal carcinoma in situ; TIL, tumor-infiltrating lymphocytes

Supplementary Table S2. Logistic regression analysis to predict high elasticity

	Univariate logistic regression					Multivariate logistic regression				
All tumor										
Predictor	OR	SE	CI_lower	CI_upper	*p*	OR	SE	CI_lower	CI_upper	*p*
Total size	1.341	0.059	1.199	1.509	<0.001	1.079	0.102	0.883	1.319	0.456
Invasive size	2.034	0.090	1.714	2.440	<0.001	1.384	0.182	0.969	1.977	0.074
DCIS %	0.992	0.003	0.986	0.998	0.006	0.994	0.008	0.980	1.009	0.466
TSR	1.004	0.003	0.998	1.010	0.164					
TIL	0.983	0.003	0.976	0.989	<0.001	0.981	0.004	0.974	0.988	<0.001
Ki67 LI	1.001	0.003	0.994	1.007	0.848					
NG	0.957	0.162	0.696	1.314	0.786					
HG	1.202	0.124	0.943	1.536	0.139					
LVI	2.349	0.164	1.708	3.247	<0.001	1.773	0.231	1.128	2.789	0.013
pT stage	3.392	0.152	2.529	4.585	<0.001	1.828	0.268	1.081	3.090	0.024
LN metastasis	1.786	0.181	1.254	2.554	0.001	0.894	0.256	0.541	1.478	0.663
EIC	0.674	0.162	0.489	0.923	0.015	1.098	0.367	0.535	2.253	0.799
HR+HER2-BC										
Predictor	OR	SE	CI_lower	CI_upper	*p*	OR	SE	CI_lower	CI_upper	*p*
Total size	1.386	0.069	1.217	1.593	<0.001	1.008	0.117	0.801	1.269	0.945
Invasive size	1.996	0.100	1.651	2.447	<0.001	1.590	0.205	1.063	2.376	0.024
DCIS %	0.993	0.003	0.986	1.000	0.040	0.998	0.005	0.989	1.007	0.647
TSR	1.003	0.003	0.997	1.009	0.330					
TIL	0.990	0.005	0.980	0.999	0.035	0.988	0.005	0.978	0.998	0.021
Ki67 LI	1.011	0.006	1.000	1.023	0.052					
NG	1.164	0.231	0.740	1.836	0.512					
HG	1.343	0.155	0.993	1.825	0.057					

LVI	2.197	0.181	1.544	3.146	<0.001	1.539	0.249	0.945	2.507	0.083
pT stage	3.274	0.174	2.344	4.630	<0.001	1.447	0.305	0.796	2.629	0.225
LN metastasis	1.805	0.199	1.226	2.675	0.003	0.972	0.272	0.571	1.656	0.916
EIC	0.704	0.184	0.489	1.008	0.057					

HER2+BC

Predictor	OR	SE	CI_lower	CI_upper	*p*	OR	SE	CI_lower	CI_upper	*p*
Total size	0.903	0.151	0.665	1.214	0.501					
Invasive size	3.687	0.351	1.943	7.792	<0.001	5.437	0.682	1.429	20.690	0.013
DCIS %	0.983	0.007	0.969	0.996	0.013	1.005	0.010	0.985	1.024	0.642
TSR	1.018	0.010	0.999	1.038	0.070					
TIL	0.984	0.006	0.972	0.996	0.010	0.977	0.008	0.961	0.993	0.004
Ki67 LI	0.994	0.011	0.973	1.015	0.549					
NG	1.390	0.400	0.636	3.063	0.410					
HG	3.856	0.472	1.605	10.430	0.004	4.183	0.536	1.464	11.959	0.008
LVI	2.100	0.513	0.788	6.041	0.148					
pT stage	2.894	0.449	1.227	7.213	0.018	0.564	0.792	0.119	2.660	0.469
LN metastasis	1.760	0.597	0.562	6.114	0.343					
EIC	0.519	0.410	0.230	1.153	0.110					

TNBC

Predictor	OR	SE	CI_lower	CI_upper	*p*	OR	SE	CI_lower	CI_upper	*p*
Total size	1.671	0.250	1.057	2.855	0.040	1.166	0.310	0.635	2.141	0.621
Invasive size	1.826	0.291	1.064	3.383	0.038	1.107	0.542	0.382	3.206	0.851
DCIS %	0.992	0.010	0.972	1.011	0.428					
TSR	1.004	0.011	0.983	1.027	0.694					
TIL	0.966	0.010	0.946	0.983	<0.001	0.961	0.011	0.940	0.983	<0.001
Ki67 LI	0.999	0.008	0.983	1.016	0.911					
NG	0.554	0.531	0.192	1.565	0.266					

HG	0.974	0.483	0.377	2.533	0.957					
LVI	3.522	0.658	1.020	14.252	0.056					
pT stage	3.965	0.502	1.513	10.964	0.006	4.018	0.894	0.697	23.156	0.120
LN metastasis	0.931	0.803	0.172	4.548	0.929					
EIC	0.608	0.575	0.187	1.835	0.387					

OR, odds ratio; SE, standard error; CI, confidence interval; HR+HER2-, hormone receptor-positive, HER2-negative; BC, breast cancer; DCIS, ductal carcinoma in situ; TSR, tumor stroma ratio; TIL, tumor-infiltrating lymphocytes; LI, labeling index; HG, histologic grade; NG, nuclear grade; LVI, lymphovascular invasion; LN, lymph node; EIC, extensive intraductal component (>25%); TNBC, triple-negative breast cancer

Supplementary Table S3. Linear regression analysis assessing factors correlated with elasticity

	Univariate linear regression					Multivariate linear regression				
All tumor										
Predictor	β	SE	CI_lower	CI_upper	*p*	β	SE	CI_lower	CI_upper	*p*
Total size	10.413	1.916	6.652	14.175	<0.001	0.275	4.786	-9.126	9.677	0.954
Invasive size	23.554	2.314	19.013	28.096	<0.001	10.150	6.707	-3.023	23.324	0.131
DCIS %	-0.445	0.103	-0.648	-0.242	<0.001	0.081	0.312	-0.533	0.694	0.796
TSR	0.209	0.106	<0.001	0.418	0.050	0.187	0.101	-0.012	0.386	0.066
TIL	-0.618	0.109	-0.832	-0.404	<0.001	-0.680	0.126	-0.928	-0.432	<0.001
Ki67 LI	0.115	0.127	-0.134	0.364	0.364					
NG (1/2 vs 3)	2.822	6.186	-9.320	14.964	0.648					
HG (I/II vs III)	11.211	7.717	-3.936	26.358	0.147					
pT stage	46.321	4.849	36.804	55.839	<0.001	22.730	9.543	3.987	41.473	0.018
LVI	29.501	5.808	18.101	40.902	<0.001	12.413	8.605	-4.489	29.314	0.150
LN metastasis	16.144	6.596	3.196	29.092	0.015	-11.782	9.280	-30.010	6.446	0.205
EIC	-21.141	5.810	-32.545	-9.737	<0.001	-8.927	13.935	-36.297	18.442	0.522
HR+HER2-										
Predictor	β	SE	CI_lower	CI_upper	*p*	β	SE	CI_lower	CI_upper	*p*
Total size	11.420	2.094	7.307	15.533	<0.001	0.517	3.758	-6.864	7.897	0.891
Invasive size	22.132	2.451	17.320	26.944	<0.001	12.939	5.829	1.492	24.386	0.027
DCIS %	-0.432	0.122	-0.672	-0.192	<0.001	-0.270	0.271	-0.803	0.263	0.321
TSR	0.145	0.116	-0.084	0.374	0.213					
TIL	-0.520	0.172	-0.857	-0.182	0.003	-0.628	0.171	-0.964	-0.291	<0.001
Ki67 LI	0.439	0.204	0.038	0.840	0.032	0.431	0.207	0.026	0.837	0.037
NG (1/2 vs 3)	9.721	9.000	-7.952	27.394	0.280					
HG (I/II vs III)	11.828	12.651	-13.016	36.673	0.350					

pT stage	44.123	5.359	33.600	54.647	<0.001	14.452	9.554	-4.310	33.215	0.131
LVI	28.624	6.331	16.191	41.056	<0.001	16.334	7.905	0.809	31.859	0.039
LN metastasis	18.831	7.074	4.939	32.724	0.008	-5.766	8.603	-22.661	11.130	0.503
EIC	-20.177	6.603	-33.143	-7.211	0.002	1.425	12.935	-23.977	26.828	0.912
HER2+BC										
Predictor	β	SE	CI_lower	CI_upper	*p*	β	SE	CI_lower	CI_upper	*p*
Total size	2.613	6.079	-9.445	14.671	0.668					
Invasive size	44.805	9.649	25.664	63.946	<0.001	29.508	19.342	-8.977	67.993	0.131
DCIS %	-0.584	0.254	-1.089	-0.079	0.024	0.329	0.328	-0.324	0.983	0.319
TSR	0.822	0.365	0.096	1.549	0.027	0.398	0.351	-0.300	1.096	0.260
TIL	-0.809	0.228	-1.261	-0.357	0.001	-0.843	0.250	-1.340	-0.346	0.001
Ki67 LI	0.101	0.429	-0.750	0.953	0.814					
NG (1/2 vs 3)	16.781	16.068	-15.093	48.655	0.299					
HG (I/II vs III)	29.298	17.934	-6.279	64.874	0.105					
pT stage	54.503	16.244	22.281	86.726	0.001	14.452	25.978	-37.237	66.140	0.580
LVI	20.988	19.724	-18.139	60.116	0.290					
LN metastasis	-3.106	23.315	-49.357	43.145	0.894					
EIC	-25.761	16.194	-57.887	6.364	0.115					
TNBC										
Predictor	β	SE	CI_lower	CI_upper	*p*	β	SE	CI_lower	CI_upper	*p*
Total size	20.387	7.466	5.497	35.277	0.008	10.257	8.802	-7.316	27.830	0.248
Invasive size	19.945	9.111	1.773	38.117	0.032	-19.919	14.157	-48.185	8.346	0.164
DCIS %	-0.117	0.333	-0.781	0.547	0.727					
TSR	0.259	0.394	-0.532	1.050	0.514					
TIL	-0.888	0.256	-1.399	-0.378	0.001	-0.736	0.236	-1.206	-0.266	0.003
Ki67 LI	0.030	0.299	-0.567	0.627	0.920					
NG (1/2 vs 3)	-9.425	19.089	-47.496	28.646	0.623					

HG (I/II vs III)	16.436	17.352	-18.172	51.043	0.347					
pT stage	58.645	15.659	27.415	89.875	<0.001	68.124	22.385	23.430	112.818	0.003
LVI	47.893	21.518	4.976	90.809	0.029	36.395	19.343	-2.225	75.015	0.064
LN metastasis	-2.096	28.908	-59.752	55.560	0.942					
EIC	-12.400	20.112	-52.513	27.713	0.540					

SE, standard error; CI, confidence interval; HR+HER2-, hormone receptor-positive, HER2-negative; BC, breast cancer; DCIS, ductal carcinoma in situ; TSR, tumor stroma ratio; TIL, tumor-infiltrating lymphocytes; LI, labeling index; HG, histologic grade; NG, nuclear grade; LVI, lymphovascular invasion; LN, lymph node; EIC, extensive intraductal component (>25%); TNBC, triple-negative breast cancer
